# Deletion Testing of the *DEGS1* Gene Should Be Part of the Diagnostic Pipeline for Hypomyelinating Leukodystrophy (HLD18)

**DOI:** 10.1155/humu/3531508

**Published:** 2025-05-07

**Authors:** Mariateresa Zanobio, Francesca Nardecchia, Gerarda Cappuccio, Maria Elena Onore, Pasquale Di Letto, Sarah Iffat Rahman, Gaetano Terrone, Lorenzo Ugga, Agnese De Giorgi, Michele Dei Cas, Marco Trinchera, Vincenzo Leuzzi, Giulio Piluso, Vincenzo Nigro, Nicola Brunetti-Pierri, TUDP consortium, Annalaura Torella

**Affiliations:** ^1^Department of Precision Medicine, University of Campania “Luigi Vanvitelli”, Naples, Italy; ^2^Department of Human Neuroscience, Sapienza University of Rome, Rome, Italy; ^3^Child Neurology, Department of Translational Medicine, Federico II University, Naples, Italy; ^4^Department of Translational Medicine, University of Naples “Federico II”, Naples, Italy; ^5^Department of Health Sciences, University of Milan, Milano, Italy; ^6^Department of Medicine and Surgery (DMC), University of Insubria, Varese, Italy; ^7^Telethon Institute of Genetics and Medicine (TIGEM), Naples, Italy; ^8^Scuola Superiore Meridionale (SSM, School of Advanced Studies), Genomics and Experimental Medicine Program, University of Naples Federico II, Naples, Italy; ^9^Fondazione Telethon, Milan, Italy

## Abstract

Hypomyelinating leukodystrophies are a heterogeneous group of disorders characterized by abnormal myelin formation in the central nervous system. Thanks to the increased use of NGS, a growing number of pathogenic single nucleotide variants in *DEGS1* have recently been reported to be responsible for hypomyelinating leukodystrophy 18 (HLD18), a rare and severe autosomal recessive form. *DEGS1* is a small gene (4 exons and 17 kb) encoding *Δ*4-dihydroceramide desaturase, which catalyzes the final step in ceramide biosynthesis. Here, we present two patients from unrelated families affected by severe and progressive white matter disease with developmental delay with or without regression and severe intellectual disability. Trio exome sequencing (ES) revealed in both probands two homozygous missense variants in the *DEGS1* gene, p.Asp16His and p.Asn255Ser, both inherited from their heterozygous healthy mothers and with a noncarrier father. This curious finding of inconsistent segregation data raises the need for further testing. There is no MLPA test available for this gene, as no deletions have been reported. However, we tried a customized high-resolution 1 M CGH array, which was surprisingly positive in both cases: a 63-kb heterozygous deletion encompassing the entire gene in one proband and a 7-kb heterozygous deletion of Exons 2–3 in the second case. Previously reported cases of HLD18 have all been found to carry single nucleotide pathogenic variants in *DEGS1*, and the two patients described here are the first to carry whole or partial microdeletions involving *DEGS1* that unmask pathogenic missense variants on the other allele. These two cases report the first examples of microdeletions of *DEGS1* that unmask recessive allele pathogenic variants, underscoring the importance of considering whole or partial gene deletions in the diagnostic pipeline.

## 1. Introduction

Hypomyelinating leukodystrophies (HLDs) are rare and genetically heterogeneous disorders characterized by abnormal myelin formation within the central nervous system (CNS). By brain magnetic resonance imaging (MRI), patients with HLDs show cerebral hypomyelination that results in progressive motor and cognitive impairment [1]. *DEGS1* was recently found to be associated with HLD. *DEGS1* gene encodes the *Δ*4-dihydroceramide desaturase that catalyzes the conversion of dihydroceramide (DhCer) into ceramide (Cer) in the final step of the biosynthesis of Cers, the backbone of all sphingolipids [2]. In the CNS, sphingolipids are particularly abundant in myelin that protects axons and permits axonal impulse conduction. Lack of proper myelination results in global impairment of CNS functioning. Pathogenic variants in *DEGS1* result in defective sphingolipid metabolism, aberrant myelination, and severe neurocognitive impairment [3]. To date, 26 patients from 17 unrelated families carrying homozygous or compound heterozygous rare single nucleotide variants (SNVs) in *DEGS1* have been reported [2–6]. Their phenotypes range from severe to mild forms, with the most severe cases characterized by severe neurocognitive impairment; neurodegeneration; hypomyelination; progressive atrophy of the corpus callosum, thalami, and cerebellum; pharmacoresistant epilepsy; developmental delay; dystonia; spasticity; and peripheral neuropathy. The milder cases presented with delayed onset and moderate cognitive regression [3–7]. Patients carrying biallelic *DEGS1* pathogenic variants showed increased DhCer/Cer ratio in serum and fibroblasts [4]. Here, we describe two patients from unrelated families enrolled in the Telethon Undiagnosed Diseases Program (TUDP) with HDL who were both found to be compound heterozygous for a missense variant and partial or whole *DEGS1* gene deletion.

## 2. Materials and Methods

The two patients were enrolled in the TUDP, and informed consent for inclusion and publication was obtained from parents. Genomic DNA was extracted from fresh peripheral blood samples using FlexiGene DNA Kit (Qiagen, Hilden, Germany), according to the manufacturer's instruction.

### 2.1. Exome Sequencing (ES)

Library preparation has been performed using SureSelectQXT Automated Target Enrichment for the Illumina Platform (Agilent Technologies, Santa Clara, California, United States), following the manufacturer's instructions. The trio WES were enriched using the SureSelect Human All Exon v7 (Agilent Technologies, Santa Clara, California, United States). The obtained libraries were assessed for quantity, using Qubit dsDNA HS Assay Kit for Qubit 3.0 fluorometer (Thermo Fisher, Massachusetts, United States), and quality using the High-Sensitivity DNA ScreenTape Assay Kit for Agilent 4200 TapeStation (Agilent Technologies, Santa Clara, California, United States). Libraries were then sequenced using the high-throughput Illumina NovaSeq 6000 system, performing paired-end runs covering at least 2 × 150 nt (Illumina Inc., San Diego, California, United States). A medium coverage of 100× is usually obtained, with 90% of the regions covered by at least 40 reads. The generated sequences were analyzed using an in-house pipeline designed to automate the analysis workflow [8].

### 2.2. Copy Number Variant (CNV) Analysis

CNV analysis was performed using a customized exome-based SurePrint G3 1 × 1M array-CGH with a full single-exon coverage, including all human genes currently associated with genetic diseases and haploinsufficient ones. Technical array-CGH was performed using SureTag Complete DNA Labeling Kit and Oligo aCGH/ChIP-on-chip Hybridization Kit (Agilent Technologies, Santa Clara, California, United States), according to the manufacturer's instruction. Data analysis was carried out using the Cytogenomics software (Agilent Technologies, Santa Clara, California, United States).

### 2.3. Sanger Sequencing

The identified pathogenic variants and the breakpoints of the *DEGS1* deletions were validated by direct Sanger sequencing using the BigDye version 3.1 sequencing kit on a 3500xl Genetic Analyzer (Applied Biosystems, Massachusetts, United States).

### 2.4. Long-Read Sequencing

For Patient 2, high molecular weight DNA was extracted from a peripheral blood sample using the PureGene Blood Core Kit (Qiagen, Hilden, Germany). The DNA was fragmented to achieve a target size range of 8–12 kb using g-TUBE (Covaris, PerkinElmer Company). The sequencing library was generated by Oxford Nanopore Technologies (ONT) Ligation Sequencing Kit (SQK-LSK114) according to the manufacturer's protocol and was sequenced on the PromethION. The generated FASTQ files were then concatenated and aligned against the hg38 reference human genome using minimap2 version 2.17-r941 employing the -x map-ont parameter specific for ONT. For a more comprehensive analysis, the EPI2ME labs human variation workflow pipeline was run on the BAM files, providing consolidated and annotated results for CNVs of the sample. Integrated Genomics Viewer (IGV) was used to visually inspect the aligned BAM files.

### 2.5. Analytical Procedures

An expression plasmid coding p.Asp16His *DEGS1* variant was obtained inserting the c.46G>C mutation in pCDNA3-DEGS1 by site directed mutagenesis and transfected into HEK-293T cells where *DEGS1* gene was previously knocked out using CRISPR-Cas9 genome editing, as previously described [9]. The homogenate of transfected cells was used as the enzyme source for measuring DEGS1 activity toward deuterated DhCer in an assay based on the detection of deuterated Cer by LC-MS/MS. Procedures and kinetic calculations were performed as recently reported [10].

Circulating sphingolipid was extracted with methanol/chloroform mixture (850 *μ*L, 2:1, *v*/*v*) from diluted plasma (100 *μ*L, 1:4). Then, prevalent interfering lipids (phospholipids and triacyclglycerols) were depleted by alkaline methanolysis (75 *μ*L KOH 1 M, 2 h at 38°C). After neutralization and centrifugation (15 min at 15,000×*g*), the organic phase was evaporated under a stream of nitrogen. The residues were dissolved in 100 *μ*L of methanol + 0.5 mg/mL butylhydroxytoluene and withdrawn in a glass vial. The LC-MS/MS consisted of a LC Dionex 3000 UltiMate (Thermo Fisher Scientific, Waltham, Massachusetts, United States) coupled to a tandem mass spectrometer AB Sciex 3200 QTRAP (AB Sciex, Concord, Canada) equipped with electrospray ionization TurboIonSpray source operating in positive mode (ESI+). Details can be found in [9].

### 2.6. Three-Dimensional Homology Modeling

Using the available model of DEGS1 enzyme predicted by AlphaFold (AF-O15121-F1.pdb), the 3D structure of the wild-type and mutated protein was obtained using DynaMut2 [11, 12].

## 3. Results

### 3.1. Case Presentation

#### 3.1.1. Proband 1

**Figure 1:**
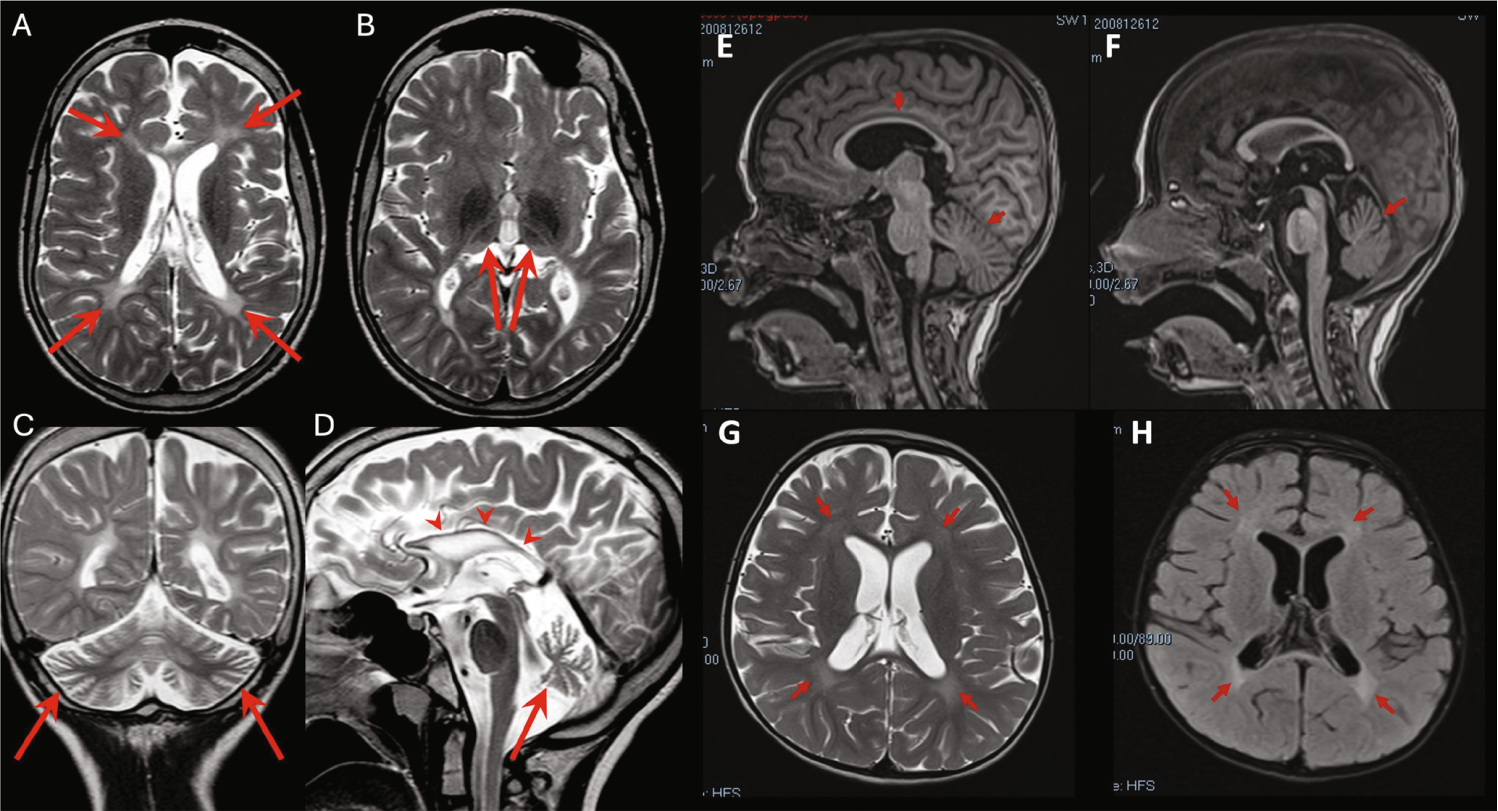
In the left panel, brain MRI scan of affected proband of Family 1; axial (A, B), coronal (C), and sagittal (D) T2-weighted sequences showing volumetric reduction and hyperintensity of periventricular and deep white matter (arrows in A). The thalami are atrophic and show contextual laminar hyperintensity (arrows in B). Hemispheric and vermian cerebellar atrophy is also present (arrows in C and D), as well as a markedly thinned corpus callosum (arrowheads in D). In the right panel, brain MRI scan of affected proband of Family 2; T1-weighted sagittal image (E, F), T2-weighted axial image (G), and FLAIR axial image (H) show mild dilatation of the lateral ventricles with diffuse hypomyelination, more pronounced in the frontal and occipital periventricular white matter (arrows in G and H), thinning of the corpus callosum (arrow in E), and mild cerebellar atrophy (arrows in E and F).

The 17-year-old boy was the firstborn of nonconsanguineous healthy parents. He had an unaffected sibling, but family history was remarkable for two paternal aunts affected by early-onset multiple sclerosis and a paternal first cousin with a diagnosis of amyotrophic lateral sclerosis. At birth, his weight was 2900 g and perinatal events were unremarkable. However, hypotonia and poor suction were noted from the first months of life. At 11 months, the first brain MRI showed diffuse supratentorial white matter demyelination. At 17 months, he starts to experience focal seizures characterized by oculocephalic deviation, increased muscle tone, and loss of awareness. Epilepsy was drug-resistant, and at last evaluation, he was treated with levetiracetam, carbamazepine, and lacosamide with acceptable control of seizures. EEG showed multifocal epileptic abnormalities over the frontal–temporal leads of the right hemisphere increased by sleep. At 3 years, he underwent gastrostomy tube insertion because of severe dysphagia and malnutrition. He progressively developed limb spasticity and was treated with botulinum toxin injections. A repeated brain MRI, performed at 13 years of age, detected diffuse supratentorial hypomyelination, associated with severe atrophy of both thalami, hippocampi, and cerebellar vermis and hemispheres (Figures 1A, 1B, 1C, and 1D).

At this age, neurological exam revealed facial hypomimia with open mouth, sialorrhea, severe axial hypotonia, upper and lower limb spasticity, brisk tendon reflexes, bilateral Babinski sign, and ankle clonus. Expressive language was absent and social interaction was poor. His weight is 23 kg (< 5° pc, *z*‐score = −4.38), his length 117.2 cm (< 5° pc, *z*‐score = −5), and his head circumference 51.5 cm (1°pc). An extensive metabolic workup and medium-resolution CGH array were negative.

#### 3.1.2. Proband 2

This 5.3-year-old girl was the only child of a nonconsanguineous couple. Her father had a history of generalized epilepsy in childhood under pharmacological treatment up to the age of 16. Her mother suffered from substance use disorder and assumed methadone and cannabinoids during pregnancy. Delivery was uncomplicated, and at birth, the girl showed diffuse tremors and increased concentrations of cannabinoids in plasma. She acquired head control at 3 months, trunk control at 8 months, and independent standing at 11 months. At about 1 year of age, following two intercurrent febrile episodes, she experienced neurological deterioration leading to loss of the acquired intentional and postural skills. By her first brain MRI scan, at the age of 16 months, hypomyelination in the areas of terminal myelination was detected. On physical exam, at the age of 35 months, the girl showed severe hypokinesia and hypomimia, trunk hypotonia and dystonia, and lower limb spasticity (scissoring, brisk tendon reflexes, clonus). Intentional movements were extremely slow and induced dystonic posturing of hands and overflow of dystonia to the mouth (spasmodic opening). Eye movements were unaffected. The girl showed good social interest and interaction. At the age of 30 months, repeated brain MRI confirmed cerebral hypomyelination (Figures 1E, 1F, 1G, and 1H). Ophthalmologic examination, abdominal ultrasound, and echocardiogram were all unremarkable. An extensive metabolic workup was unconclusive. Cerebrospinal fluid (CSF) analysis of amino acids, neurotransmitters, pterins 5-methyltratrahydrofolate, and lactate was all normal. Glycorrhachia was mildly decreased (2.8 mmol/L, normal values: 3.3–4.4) but sequencing of *SLC2A1* did not show any alteration. Symptomatic treatments such as baclofen and trihexyphenidyl resulted in little or no improvement despite side-effects. A carbidopa–levodopa trial (slowly titrated up to 5 mg/kg/die) induced a transient improvement of the motor control and intestinal transit. However, this drug was discontinued because of poor compliance.

### 3.2. Molecular and Functional Data

**Figure 2:**
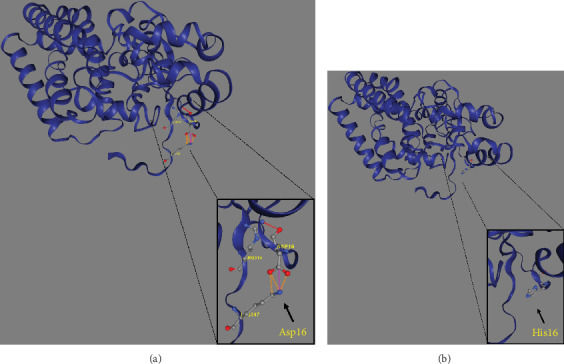
3D modeling of the wild-type (a) and mutant (b) DEGS1 protein using DynaMut2 online tool. Black arrows indicate the stabilizing bond between Arg314 and Lys317 at the C-terminus of the protein and Asp16. The zoomed-in figure within the rectangle of (b) demonstrated that this bond is disrupted due to the substitution of Asp16 with histidine.

**Figure 3:**
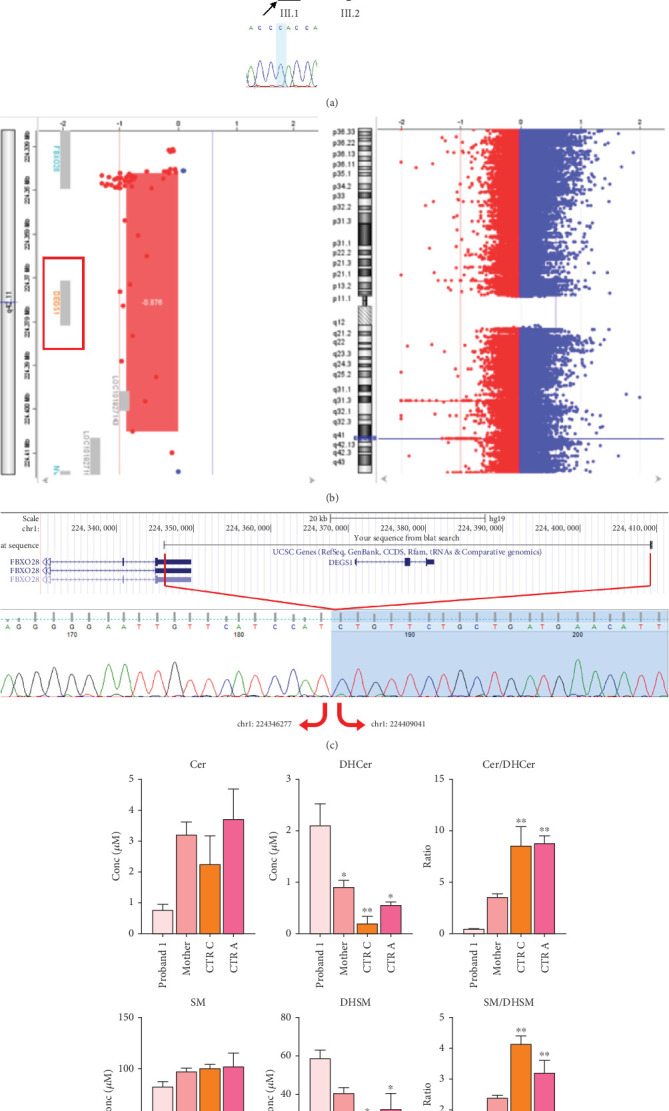
Family 1 pedigree with Sanger sequencing data of causative SNV in *DEGS1* gene (a); results of custom CGH array from Cytogenomics software indicating the deletion of *DEGS1* gene (b); Sanger sequencing data of breakpoints in *DEGS1* gene (c). Ratio of unsaturated/saturated sphingolipids (Cer/DHCer and SM/DHSM) determined in the serum of Proband 1, his mother, two age-matched healthy controls (CTR C), and five healthy adult controls (CTR A), as determined by LC-MS/MS; statistical analysis was performed using one-way ANOVA coupled to Bonferroni post hoc test for multiple comparison (d).

Trio ES was performed in both probands and their parents. ES data in Proband 1 showed the nucleotide variant NM_003676.4:c.46G>C in the first exon of *DEGS1*, resulting in the not previously reported p.Asp16His amino acid substitution. The variant is classified as likely pathogenic (PM2, PM3, PS3) according to ACMG guidelines. This variant was absent in gnomADv4.0, ExAC, 1000 Genomes, or dbSNP155 database, and it was predicted to be deleterious by several pathogenicity prediction tools (see Table S1). Using 3D homology modeling, this amino acid substitution appears to modify the intramolecular interactions of the DEGS1 protein. In the wild type, the Asp16 residue stabilizes the C-terminus of the protein through its binding to Arg314 and Lys317 (Figure 2a). In the mutant, the His16 residue loses these interactions, resulting in a possibly slightly destabilized conformation of the enzyme (Figure 2b). Proband 1 was apparently homozygous for the c.46G>C variant, with only his mother being heterozygous (Figure 3a). A high-resolution custom CGH array identified a paternally inherited deletion of approximately 63 kb encompassing the entire *DEGS1* gene and precise breakpoint localization was defined by Sanger sequencing (Figure 3b,c). The identified deletion spanned a region of 62,765 bp on chromosome 1 (chr1:224,346,277-224,409,041).

**Figure 4:**
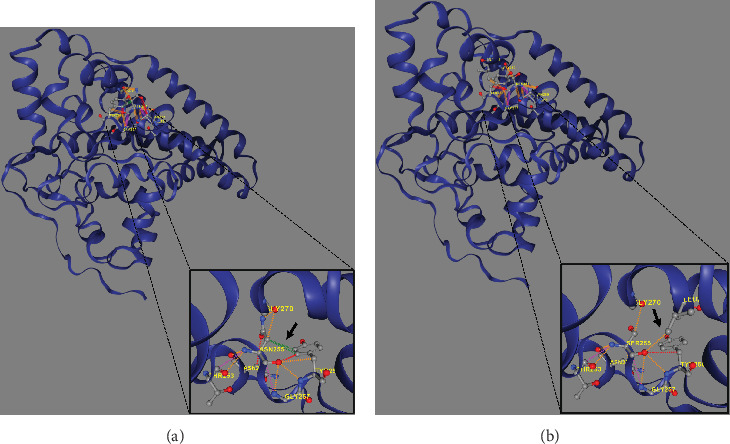
3D modeling of the wild-type (a) and mutant (b) DEGS1 protein using the DynaMut2 online tool. Black arrows indicate the loss of physiological hydrophobic bonds between Asn255 and the aromatic ring of Tyr258 (green dotted line) as well as the newly formed polar bond between Ser255 and Leu273 (orange dotted line), due to amino acid substitution. These intramolecular rearrangements alter the proper folding of the enzyme and may negatively impact its function.

**Figure 5:**
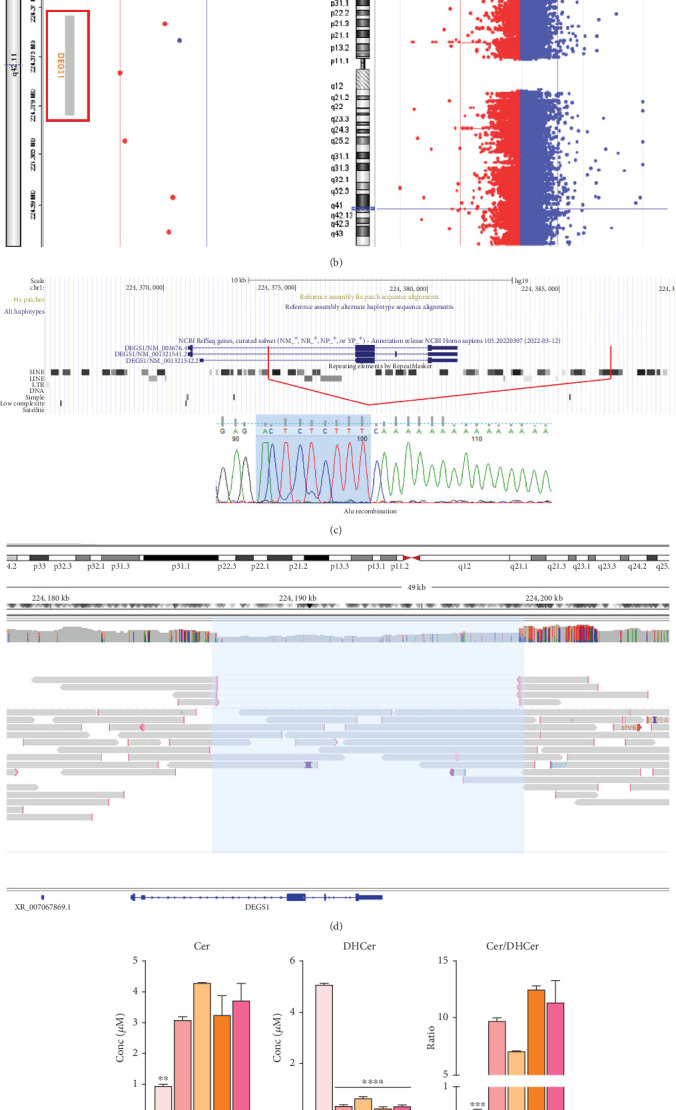
Family 2 pedigree with Sanger sequencing data of causative SNV in *DEGS1* gene (a). Results of custom CGH array from Cytogenomics software indicating the deletion of *DEGS1* gene (b). Sanger sequencing data of breakpoints in *DEGS1* gene (c). IGV screen of LR-WGS by ONT, showing heterozygous deletion of *DEGS1* (d). Ratio of unsaturated/saturated sphingolipids (Cer/DHCer and SM/DHSM) determined in RM1073 proband, her mother and father, and two age-matched healthy controls (CTR C) and five healthy adult controls (CTR A). Statistical analysis was performed using one-way ANOVA coupled with Bonferroni post hoc test for multiple comparison (e).

Trio ES in Proband 2 showed the already reported pathogenic variant (PM2, PM3, PS3, PS4, PP5 according to ACMG guidelines) NM_003676.4:c.764A>G in the Exon 2 of *DEGS1*, resulting in the p.Asn255Ser amino acid substitution that affects a highly conserved residue lying in the *Δ*4-sphingolipid-FADS-like domain, essential for the enzyme activity [13]. Consistently, 3D homology modeling of mutant DEGS1 revealed changes in the intramolecular bonds induced by the p.Asn255Ser substitution. Specifically, the hydrophobic bonds between Asn255 and the aromatic ring of Tyr258 are lost in the presence of Ser255 and a new polar bond with Leu273 is created. These changes in the proper folding of the enzyme can negatively affect its function (Figure 4a,b). As in Family 1, this proband also appeared to be homozygous for the missense variant, with only his mother being heterozygous. CNV analysis showed a ~7 kb deletion encompassing Exons 2 and 3 of *DEGS1* on the paternal allele (Figure 5a,b). Sanger sequencing performed on this locus unraveled two short interspersed nuclear elements (SINEs) of the AluY families at both the 5⁣′- and the 3⁣′-end of the deleted region (Figure 5c), which potentially drive the recombination causing the genomic rearrangement. This result was confirmed with whole genome long-read sequencing that clearly detected the deleted region with the flanking SINEs (Figure 5d).

Functional studies were carried out to verify the pathogenicity of the identified variants and to assess their impact on DEGS1 activity. When expressed in cells lacking DEGS1 activity, the p.Asp16His *DEGS1* variant had reduced activity compared to the wild-type enzyme [10], with lower affinity for the substrate (Km 5.48 mM vs. 2.41 of WT), much lower Vmax (4.57 pmol/mg × min vs. 16.5 of WT), and overall catalytic efficiency (Km/Vmax 1.19 vs. 6.85 of WT). Consistent with the reduced enzyme activity, in the proband's serum, saturated Cer and SM were higher than the corresponding unsaturated forms and the Cer/DhCer and SM/DhSM ratios were increased in the probands compared to controls (Figure 3d).

Transfection of *DEGS1* knocked out cells with a plasmid carrying the *DEGS1* p.Asn255Ser variant also resulted in reduced protein and desaturase activity and reduced Cer/DhCer and sphingomyelin/dihydrosphingomyelin ratios, as previously shown [9]. In the proband's serum saturated Cer and SM were equal or higher than the corresponding unsaturated forms in contrast to controls that showed an opposite signature. In particular, the reduction of Cer and SM and the increased DhCer and DhSM resulted in greatly reduced Cer/DhCer and SM/DhSM ratios in the probands compared to controls (Figure 5). Moreover, the availability of fibroblasts from Patient 2 allowed us to verify that they had very low levels of DEGS1 protein and a low ratio between unsaturated and saturated sphingolipids, as previously reported in greater details [10].

## 4. Discussion

Here, we described two patients with HLD18 with clinical presentations largely overlapping the clinical manifestations of HLD18 cases reported so far, including failure to thrive, severe developmental delay with minimal-to-absent gain and often loss of developmental milestones, spasticity, epilepsy, and leukodystrophy (Table S2).

**Figure 6:**
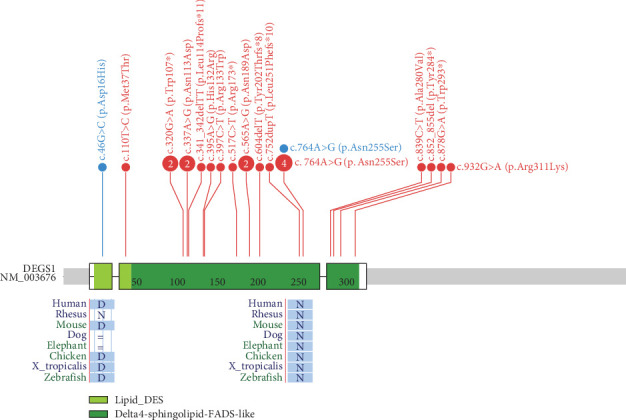
Graphical representation of variants in *DEGS1* gene by ProteinPaint (https://proteinpaint.stjude.org/) [14, 15]. In red are reported the already published family variants; in light blue are patient variants. Evolutionary conservation sites are reported for the two missense variants identified in the two cases reported here.

In contrast to all 26 cases reported so far, all carried *DEGS1* homozygous or compound heterozygous rare SNVs (Figure 6), our two cases carrying whole or partial deletion of the *DEGS1* gene in combination with a missense variant. The p.Asp16His variant found in Family 1 has not been previously reported and its catalytic properties are impaired *in vitro* as much as those reported for other missense variants including that identified in Family 2. The 3D structure of wild-type and mutant DEGS1 protein obtained for both the missense variants showed modifications of intramolecular amino acid bonds, leading to different secondary structure, predicted to interfere with protein folding and function. Consistently, serum sphingolipid profile in both probands showed increased Cer/DhCer ratio, thus providing functional confirmation of the biochemical defect. The p.Asn255Arg substitution identified in Family 2 was present in 4 out of the 17 cases reported so far, both in homozygous and compound heterozygous states [4, 5]. The relatively high frequency in patients from different geographic areas suggests that this variant occurs in a potential mutational hot spot.

Using a high-resolution CGH array, we detected two distinct deletions of *DEGS1* gene on the paternally inherited alleles: one encompassing the whole gene (Family 1) and the second involving the Exons 2 and 3 (Family 2). No overlapping CNVs were found in the Database of Genomic Variants (DGVs) [16], supporting the rarity and the potential pathogenicity of these variations. Notably, in both of our patients, the CNVs identified in *DEGS1* spanned several kilobases in length, with breakpoints occurring in intronic or UTR regions, which are less thoroughly covered in ES. Therefore, the identified medium- to large-scale deletions, along with the locations of breakpoints, mark a limitation in their detection using ES data. Interestingly, the presence of the AluY repeated elements detected at both 5⁣′- and 3⁣′-end of the deleted region, in Patient 2, suggests an Alu-mediated recombination process leading to *DEGS1* microdeletion. The physiological presence of these SINEs could represent a noninfrequent mechanism underlying intergenic deletions [17]. However, the prevalence of *DEGS1* microdeletion as genetic lesion responsible for HLD18 remains to be determined.

*DEGS1* encodes an enzyme responsible for the final step of the biosynthesis of Cers [2], and human cellular models showed that both complete and partial *DEGS1* loss of function variants affect cellular ratio of sphingolipids and dihydrosphingolipids [9]. Changes in the serum concentrations of unsaturated/saturated sphingolipids have been observed also in the parents of the affected patients heterozygous for *DEGS1* pathogenic variants. Interestingly, three members of Family 1 developed in adulthood a neurologic disease that received the clinical diagnoses of multiple sclerosis or amyotrophic lateral sclerosis. Unfortunately, they were not available for genetic testing, but this family history leads to speculating that a single hit in *DEGS1* could be a risk factor for these neurological disorders. The pathogenesis of HLD18 is not understood, but it has been proposed that DEGS1 deficiency results in the formation of aberrant and potentially neurotoxic sphingosine metabolites upstream of the DEGS1 substrate [3]. If this hypothesis holds true, a substrate reduction therapy would be expected to reduce the accumulation of toxic metabolites and to provide clinical benefit.

In conclusion, we report two patients with severe and progressive white matter disease and presenting with developmental delay with or without regression and severe intellectual disability evaluated by integrated SNV and CNV analyses that revealed partial or whole gene deletions unmasking missense variants in *DEGS1*.

## Data Availability

Additional data are available from the corresponding author on reasonable request.

## Supporting Information

Supporting Information Additional supporting information can be found online in the Supporting Information section. Table S1: Pathogenic prediction for SNVs in *DEGS1*. Table S2: Genetic and clinical findings of individuals with causative variants in *DEGS1*.
