# Supplementary material for: Deletion Testing of the DEGS1 Gene Should Be Part of the Diagnostic Pipeline for Hypomyelinating Leukodystrophy (HLD18)
Source: Hum Mutat. 2025 May 7;2025:3531508. doi: 10.1155/humu/3531508 (PMC12077970; doi:10.1155/humu/3531508)
Supplement: Supporting Information — Additional supporting information can be found online in the Supporting Information section. Table S1: Pathogenic prediction for SNVs in DEGS1. Table S2: Genetic and clinical findings of individuals with causative variants in DEGS1. [file 3531508.f1.docx]

Supplementary Table 1. Pathogenic prediction for SNVs in *DEGS1*

| *PREDICTION TOOLS* | *VARIANTS* | |
| --- | --- | --- |
|  | **NM_003676.4:c.46G>C (SNV Family 1)** | **NM_003676.4:c.764A>G (SNV Family 2)** |
| CADD | 27.1 | 24.0 |
| Mutation assessor | Pathogenic Supporting (score=3.325) | Pathogenic Supporting (score=3.285) |
| SIFT | Pathogenic Supporting (score=0.001) | Benign Supporting (score=0.032) |
| M-CAP | Pathogenic Moderate (score=0.7774) | Benign Supporting (score=0.05151) |
| EVE | Pathogenic Supporting (score=0.688) | Pathogenic Strong (score=0.9165) |
| LRT | Pathogenic Supporting (score=0) | Pathogenic Supporting score=0) |

Supplementary Table 2. Genetic and clinical findings of individuals with causative variants in *DEGS1*

|  |  | Cases of the present study | | | | Devesh C. Pant et al. 2019 | | | | | |
| --- | --- | --- | --- | --- | --- | --- | --- | --- | --- | --- | --- |
|  | **Total cases** | **Famlily 1** | | **Family 2** | | **Family 1** | | **Family 2** | | **Family 3** | |
| *DEGS1* VARIANT INFORMATION |  |  |  |  |  |  |  |  |  |  |  |
| Inheritance | Comp Het=7; Hom=12 | Comp Het | | Comp Het | | Hom | | Comp Het | | Comp Het | |
| Variant annotation (NM_003676.4, NC_000001.10, NP_003667.1) | Missense=16; StopGain=7; IntergenicDeletion=2 | c.46G>C, p.Asp16His | c.-97_*963del, p.? | c.764A>G, p.Asn255Ser | c.83-434_*963del, p.? | c.395A>G, p.His132Arg | | c.397C>T, p.Arg133Trp | c.752dup, Leu251Phefs*10 | c.341_342del, p.Leu114Profs*11 | c.764A>G, p.Asn255Ser |
| *PATIENT INFORMATION* |  | Proband 1 | | Proband 2 | | Patient 1 | Patient 2 | Patient 3 | | Patient 4 | Patient 5 |
| Sex | M=16; F=12 | M | | F | | M | M | F | | F | M |
| Ethnicity |  | European | | European | | Pakistani | | European descent | | European descent | |
| Age of onset (months) | average= 5,6 | 6 | | 12 | | 2 | 1 | 12 | | 6 | 6 |
| Language delay | 26/26 | + | | + | | + | + | + | | + | + |
| Delayed motor development | 23/24 | + | | + | | + | + | NA | | + | - |
| Intellectual disability | 17/19 | + | | + | | + | + | NA | | NA | - |
| Regression | 8/27 | - | | + | | - | - | + | | - | - |
| Seizures | 19/26 | + | | - | | - | - | + | | + | + |
| Dystonia/Spasticity | 23/28 | + | | + | | + | + | + | | + | + |
| Failure to thrive | 15/21 | + | | + | | + | + | + | | + | + |
| Acquired microcephalia (SD) | 7/21 | + | | + | | - | + | - | | - | - |
| White Matter Disease | 25/25 | + | | + | | + | + | + | | + | + |
| Abbreviations: Comp Het: Compound Heterozygous; Hom: Homozygous; NA: not available | | | | | | | | | | | |

Supplementary Table 2. Genetic and clinical findings of individuals with causative variants in *DEGS1* (continue)

|  | Devesh C. Pant et al. 2019 | | | | | | | | | | | |
| --- | --- | --- | --- | --- | --- | --- | --- | --- | --- | --- | --- | --- |
|  | **Family 4** | **Family 5** | | **Family 6** | **Family 7** | **Family 8** | | | **Family 9** | | **Family 10** | |
| *DEGS1* VARIANT INFORMATION |  |  |  |  |  |  |  |  |  |  |  |  |
| Inheritance | Hom | Hom | | Hom | Hom | Hom | | | Comp Het | | Hom | |
| Variant annotation (NM_003676.4, NC_000001.10, NP_003667.1) | c.604del, p.Tyr202Thrfs*8 | c.764A>G, p.Asn255Ser | | c.337A>G, p.Asn113Asp | c.337A>G, p.Asn113Asp | c.320G>A, p.Trp107* | | | c.565A>G, p.Asn189Asp | c.764A>G, p.Asn255Ser | c.852_855del, p.Tyr284_Asp285delins* | |
| PATIENT INFORMATION | Patient 6 | Patient 7 | Patient 8 | Patient 9 | Patient 10 | Patient 11 | Patient 12 | Patient 13 | Patient 14 | | Patient 15 | Patient 16 |
| Sex | F | M | M | F | M | F | M | M | M | | M | M |
| Ethnicity | Moroccan | Moroccan | | Algerian | Algerian | Egyptian | | | Indian | | European descent | |
| Age of onset (months) | 1 | 24 | 24 | 1 | 1 | 1 | 1 | 1 | 4 | | 4 | 5 |
| Language delay | + | + | + | + | + | + | + | + | NA | | + | + |
| Delayed motor development | + | + | + | NA | + | + | + | + | NA | | + | + |
| Intellectual disability | NA | NA | NA | + | + | + | + | + | NA | | + | + |
| Regression | - | + | - | - | - | - | - | - | + | | - | - |
| Seizures | + | - | + | + | + | + | + | + | + | | - | + |
| Dystonia/Spasticity | + | + | + | + | + | + | + | + | + | | + | + |
| Failure to thrive | NA | - | - | + | + | + | + | - | + | | - | - |
| Acquired microcephalia (SD) | - | - | - | - | - | - | - | - | NA | | - | - |
| White Matter Disease | + | + | + | + | + | + | + | + | + | | + | + |
| Abbreviations: Comp Het: Compound Heterozygous; Hom: Homozygous; NA: not available | | | | | | | | | | | | |

Supplementary Table 2. Genetic and clinical findings of individuals with causative variants in *DEGS1* (continue)

|  | Devesh C. Pant et al. 2019 | | | | Gergely Karsai et al. 2019 | Vadim Dolgin et al. 2019 | | | | Huifang Yan et al. 2021 | | Melissa Song Ting Wong et al. 2023 |
| --- | --- | --- | --- | --- | --- | --- | --- | --- | --- | --- | --- | --- |
|  | **Family 11** | | **Family 12** | **Family 13** | **Family 1** | **Family 1** | | | | **Family 1** | | **Family 1** |
| *DEGS1* VARIANT INFORMATION |  |  |  |  |  |  | | | |  |  |  |
| Inheritance | Comp Het | | Hom | Hom | Hom | Hom | | | | Comp Het | | Hom |
| Variant annotation (NM_003676.4, NC_000001.10, NP_003667.1) | c.110T>C, p.Met37Thr | c.878G>A, p.Trp293* | c.320G>A, p.Trp107* | c.517C>T, p.Arg173* | c.839C>T, p.Ala280Val | c.764A>G, p.Asn255Ser | | | | c.110T>C, p.Met37Thr | c.770G>A, p.Gly257Asp | c.565A>G, p.Asn189Asp |
| PATIENT INFORMATION | Patient 17 | | Patient 18 | Patient 19 | Patient 20 | Patient 21 | Patient 22 | Patient 23 | Patient 24 | Patient 25 | | Patient 26 |
| Sex | F | | F | M | M | M | M | F | F | F | | F |
| Ethnicity | Chinese | | Egyptian | Iranian-Lor | Turkey | Arab Israeli | | | | NA | | South Asian origin |
| Age of onset (months) | 4 | | 1 | 3 | 6 | NA | NA | NA | NA | NA | | 4 |
| Language delay | + | | + | + | + | + | + | + | + | NA | | + |
| Delayed motor development | + | | + | + | + | + | + | NA | + | + | | + |
| Intellectual disability | + | | + | + | + | - | NA | NA | NA | + | | + |
| Regression | - | | - | - | NA | + | + | + | + | - | | - |
| Seizures | - | | + | - | + | NA | + | + | + | NA | | + |
| Dystonia/Spasticity | + | | + | + | + | - | - | - | - | - | | + |
| Failure to thrive | - | | + | + | NA | NA | NA | NA | NA | NA | | + |
| Acquired microcephalia (SD) | NA | | + | + | + | NA | NA | NA | NA | NA | | + |
| White Matter Disease | + | | NA | + | + | + | + | NA | NA | + | | + |
| Abbreviations: Comp Het: Compound Heterozygous; Hom: Homozygous; NA: not available | | | | | | | | | | | | |
